# Autophagy and Extracellular Vesicles in Colorectal Cancer: Interactions and Common Actors?

**DOI:** 10.3390/cancers13051039

**Published:** 2021-03-02

**Authors:** Clément Auger, Niki Christou, Aude Brunel, Aurélie Perraud, Mireille Verdier

**Affiliations:** 1 EA 3842, CAPTuR, GEIST, Faculty of Medicine, University of Limoges, 2 rue du Dr Marcland, 87025 Limoges CEDEX, France; clement.auger@unilim.fr (C.A.); aude.brunel@unilim.fr (A.B.); aurelie.perraud@unilim.fr (A.P.); mireille.verdier@unilim.fr (M.V.); 2 Endocrine, General and Digestive Surgery Department, Limoges University Hospital, 2 rue Martin Luther King, 87042 Limoges CEDEX, France

**Keywords:** autophagy, extracellular vesicles, secretory autophagy, rab-GTPases, colorectal cancer

## Abstract

**Simple Summary:**

Cancer stem cells (CSCs) are known for their ability to survive under stressful conditions. To this aim, they use autophagy to recycle their altered organelles and proteins by addressing them toward a lysosome for their degradation. CSCs can also communicate with their environment using the secretion of extracellular vesicles (EVs) which carry information, strengthening their ability to survive under such conditions. Both mechanisms are known for using common actors and have been described as implicated in colorectal cancer (CRC). However, CSCs remain difficult to target due to the lack of specific markers identified, especially in colorectal cancer. Then, the study of the crosstalk between autophagy and the secretion of EVs seems crucial regarding a better targeting of CSCs.

**Abstract:**

Autophagy is a homeostatic process involved in the degradation of disabled proteins and organelles using lysosomes. This mechanism requires the recruitment of specialized proteins for vesicle trafficking, that may also be involved in other types of machinery such as the biogenesis and secretion of extracellular vesicles (EVs), and particularly small EVs called exosomes. Among these proteins, Rab-GTPases may operate in both pathways, thus representing an interesting avenue for further study regarding the interaction between autophagy and extracellular vesicle machinery. Both mechanisms are involved in the development of colorectal cancer (CRC), particularly in cancer stem cell (CSC) survival and communication, although they are not specific to CRC or CSCs. This highlights the importance of studying the crosstalk between autophagy and EVs biogenesis and release.

## 1. Introduction

Macroautophagy, mostly known as autophagy, is a highly conserved process by which damaged proteins and organelles are recycled by a lysosome-dependent degradation, contributing to the maintenance of cell homeostasis [1]. Although cells have a basal autophagy level that maintains their equilibrium, stress conditions such as hypoxia, starvation or DNA damage are known to increase the levels of autophagy in cells [2,3]. However, this equilibrium remains fragile, and defects in autophagy machinery can contribute to malignant transformation [4]. This process is of growing interest in cancer research, with more than 15,000 publications related to autophagy in cancer published over the past ten years. As an example, autophagy helps cancer stem cells (CSCs) to survive under drastic conditions [5] and is also involved in metastasis development [6,7,8]. Among cancers, autophagy has been shown to be implicated in the development of colorectal cancer (CRC) [9,10,11]. An emerging role of autophagy in secretory mechanisms has been reported as suggesting overlaps with vesicular trafficking [12,13].

Among the mechanisms involving vesicular trafficking, the secretion of extracellular vesicles (EVs) seems to partly overlap with autophagosome formation and fate [12,13]. EVs are used by cells to communicate with their environment and the surrounding cells by carrying information such as lipids, proteins, or nucleic acids [14,15,16,17]. The “EVs” term is currently used to refer to exosomes, microvesicles and apoptotic bodies—the three main kinds of EV notably implicated in cell-to-cell communication, which differ from each other by size and origin [15,18,19]. Exosomes are a unique population due to their endosome-based origin, which is mainly regulated by the endosomal sorting complexes required for transport (ESCRT) machinery that generates multi-vesicular bodies (MVBs) that fuse with the plasma membrane to release exosomes [18,19,20,21,22,23,24,25,26,27]. MVBs can be generated by other means, and their fusion with the cell membrane involves Rab-GTPases and SNAREs protein families [14,26,28,29,30,31,32,33,34,35]. Furthermore, due to their intercellular communication abilities, EVs are of growing interest in the field of cancer research due to their implication in tumour aggressiveness and metastatic invasion, in particular in the case of CRC [36,37,38,39,40,41,42]. As potential common actors in both pathways, Rab-GTPases proteins are known to be involved in vesicular trafficking and transport, as well as autophagy-related processes.

In this review we will describe the roles of autophagy and EVs in colorectal cancer, and how these interact within the framework of cancer stem cell survival and communication, and how these may offer new insights into cancer diagnosis.

## 2. Macroautophagy

### 2.1. Introducing the Process and Its Main Actors

Macroautophagy (hereafter referred to as autophagy) is a physiological process that helps maintain cell homeostasis by recycling damaged proteins and organelles, and older or misfolded proteins, using a lysosomal degradation [1]. Autophagy thus represents a complementary mechanism of the ubiquitin-proteasome degradative machinery that specifically recognizes ubiquitin-tagged structures [43].

Autophagy initiation consists in the formation of a phagophore (nascent isolation membrane), which is engaged by the activation of the Unc-51-like kinase (ULK) complex, composed of ULK1/2, FIP200, ATG13 and ATG101. A second complex then erupts to maintain the phagophore nucleation: the class III PI3K complex, composed of Beclin-1, ATG14L and the VPS34 PI3K, which enhances the production of phosphatidylinositol-3-phosphate (PI3P) recruited to the phagophore, with the help of ATG9 [5,44]. Once the phagophore starts to expand, the ATG12-ATG5-ATG16L1 complex is recruited to pursue its maturation to become a double-membrane enclosed organelle called autophagosome. More precisely, this complex contributes to the recruitment of the microtubule-associated protein light chain B-phosphatidylethanolamine (LC3B-PE) to the autophagosomal membrane. Pro-LC3B is previously converted into LC3B-I by action of ATG4, then into LC3B-II after lipidation (PE addition) by coupled intervention of ATG3 and ATG7, mandatory for the autophagosome elongation and closure [45,46]. Furthermore, thanks to its LC3B-Interacting Regions (LIR; [47]), LC3B recognizes autophagy substrates and cargos to be loaded into the phagophore. Because it is a main actor in this process, being located in autophagosomes and on their membrane, LC3B-I/II conversion is often used as an autophagosome-closure marker.

The final step of the canonical autophagy mechanism requires the fusion of the newly formed autophagosome with a lysosome, giving rise to an autophagolysosome (or autolysosome). This step notably involves Rab-GTPases-family proteins such as Rab7, and SNARE-family proteins such as syntaxin 17 (STX17), VAMP8 and SNAP29, that help the autophagosome-lysosome encounter and fusion [48]. The cargo is then degraded following a typical lysosomal lysis.

In physiological conditions, the ULK complex is regulated by the mammalian target of rapamycin complex 1 (mTORC1) that phosphorylates ULK1/2 and ATG13, leading to their inactivation. Under stress conditions such as hypoxia, starvation or DNA damage, mTORC1 activity is decreased as a result of the PI3K/AKT pathway mitigation, and the activation of the ULK complex [2,3,49]. The drop in mTORC1 activity is usually associated with increased AMP-activated protein kinase (AMPK) activity, which also activates ULK1 and the following autophagy machinery [50,51].

### 2.2. Autophagy’s Double-Faceted Role in Carcinogenesis

As described above, autophagy is mandatory for maintaining cell integrity by its basal recycling activity and by promoting resistance to various stress conditions. Autophagy was initially believed to play an exclusively protective role, since defects led to genome instability [52] and led to the development of disease processes such as cancer [53,54]. Additionally, in early tumorigenesis stages, autophagy seems to play a tumour-suppressing role, since it prevents hypoxia-induced necrosis and inflammation, thus decreasing tumour cell proliferation [55].

Although autophagy can prevent tumour cell proliferation in early tumorigenesis steps [55], it seems to act as a pro-tumour player in later stages of tumour formation [4]. Moreover, CSCs represent a particular cell population that has higher basal autophagy levels than other cancer cells, as they use it as a way to proceed to quiescence in order to survive under drastic conditions within the tumour core [56,57]. Wolf et al. [57] thus showed that inhibiting ATG4A expression led to a decrease of the CSC-like phenotype in breast cancer tumours in vivo, suggesting its importance in maintenance of CSCs. In keeping with the promotion of CSC behaviour, autophagy was also shown to enhance epithelial to mesenchymal transition (EMT) during hepatic carcinogenesis, a process by which tumour cells (and particularly CSCs) are able to migrate and then colonize other tissues by forming metastases [58]. To further survive after their detachment from their initial extracellular matrix (ECM), EMT-proceeding cells need to resist a particular kind of detachment-induced apoptosis called anoïkis [59]. To this aim, autophagy promotes cell survival towards anoïkis during EMT, thus potentiating tumour invasion and metastasis, in which it seems to play a context-dependant role [5,6,7,60]. Also, as angiogenesis is crucial for tumoral development and dissemination by providing nutrient intake within the tumour, autophagy seems to help cancer cells to survive apoptosis after anti-angiogenic treatment in glioblastoma, suggesting that the use of coupled anti-autophagic treatments may restore its efficacy [54,61]. Hence, autophagy is of great interest in cancer research as a potential target [62,63,64].

As in other cancers, autophagy displays a context-dependent role in the development of CRC following the tumour type, its stage or the actual metabolic background such as energy requirement in the early stages, or a stress response by providing cell survival in later stages [10,65]. Consistent with these observations, autophagy is induced under stress conditions such as DNA damages in CRC cells, helped by p53 with simultaneous inhibition of mTOR pathway [66]. Autophagy not only triggers cell survival and metabolism modifications in tumour cells, but also in other cells within the microenvironment such as cancer-associated fibroblasts (CAFs), which are known to be involved in cell migration during metastasis development [11,67]. Thus, targeting autophagy in CRC is of growing interest in this research field. Our group reported that its inhibition concomitantly with BDNF/TrkB signalling axis blocking constitutes a potential new therapeutic approach for CRC [9]. It was shown that the use of chloroquine diphosphate as an autophagic flux inhibitor triggered CRC cell death when combined with the chemotherapy agent 5-fluorouracil (5-FU) suggesting that autophagy also potentiates chemoresistance in CRC [68]. Thus, studying the ability of CRC cells to enhance autophagy under stress conditions may help to predict how they respond to autophagy inhibition, thus providing information on their sensitivity and the relevance of targeting autophagy [69].

### 2.3. Secretory Autophagy and Its Potential Link with Vesicular Trafficking and Secretion

As described earlier, autophagy is known to involve vesicular trafficking, leading to lysosomal degradation. However, autophagosomes may have an alternative fate to fusing with a lysosome. The relatively new concept of secretory autophagy (SA) [70] has been studied extensively over the past decade. As it was first described as a means to unconventionally secrete inflammatory cytokines such as Interleukin-1-β (IL-1β) or Interleukin-18 (IL-18), SA was then thought as being involved in inflammatory-associated processes in general [71,72]. Subsequently, further roles have been described for SA, including lysozyme secretion by Paneth cells to counter intestinal bacterial infection [73], or insulin-degrading secretion by astrocytes in Alzheimer disease [74], as well as the secretion of proteins implicated in neurodegenerative processes such as Alzheimer’s or Parkinson’s diseases [75,76]. Although SA seems to be implicated in several different mechanisms, their common factor is the secretion of cytosolic proteins which lack a signal peptide, then resulting in their incapacity to enter the conventional ER-associated secretory pathway, thereby using an unconventional secretory mechanism [70].

**Figure 1:**
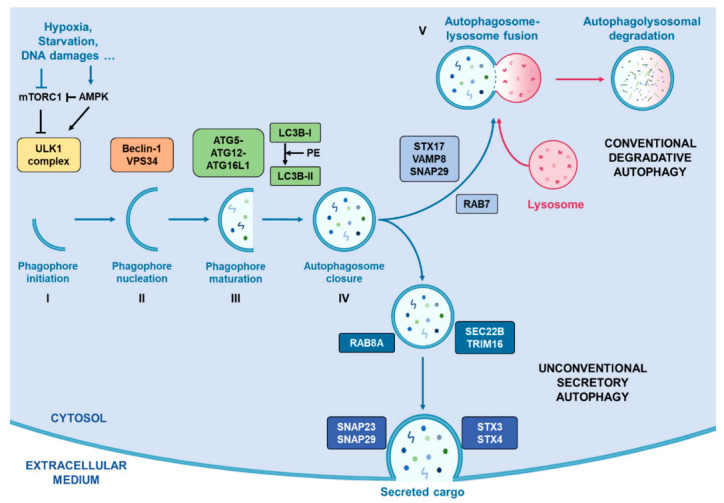
Representation of conventional degradative autophagy and unconventional secretory autophagy. Stress conditions such as hypoxia, starvation or DNA damages drive the activation of autophagic flux by inhibiting mTORC1 activity, correlating with an increased activity of AMPK, which activates the successive complexes that operate in order to initiate and complete the formation of autophagosomes. Once the autophagosome closed with the help of LC3B, two pathways exist. First, they can fuse with a lysosome with the help of RAB7, STX17, VAMP8 and SNAP29 in order to degrade its cargo. Alternatively, they can take the direction of the plasma membrane helped by RAB8A, SEC22B and TRIM16 for their transport and by SNAP23, SNAP29, SXT3 and STX4 for their fusion with the cell membrane.0.

As summarised in Figure 1, this secretory pathway involves several vesicle trafficking-related proteins such as Rab-GTPases (Rab8A) [77], and SNAREs (SEC22B) [78], that mediate SA by avoiding classical autophagosome-lysosome fusion and drive the autophagosome to fuse with the cell membrane. Indeed, unlike classical autophagy degradation, the SA pathway does not involve STX17 and its complex to guide autophagosome towards the lysosome for their fusion. Instead, the cargo receptor TRIM16 is recruited and interacts with SEC22B, thus forming a complex which drives the autophagosome towards the plasma membrane where other SNAREs (SNAP23, SNAP29, STX3 and STX4) proceed to their fusion [78].

Autophagosomes can also fuse with MVBs to form amphisomes which, along with autophagy, can follow a context-dependent fate. Indeed, amphisomes were described as being part of a classical autophagic degradation pathway in which they could fuse with a lysosome to recycle MVBs in order to avoid EVs releasing [13,79]. However, other functions have also been attributed to amphisomes, since they were shown to fuse with the plasma membrane in secretory processes in which ATG and Rab-GTPases proteins were involved, suggesting a potential overlap between SA, amphisomes and EVs releasing [12,13].

Thus, secretory autophagy is of growing interest in the study of intracellular vesicular trafficking interactions, with a number of recent studies addressing this possibility [13,14,28,70,80]. Despite its increasing significance, the role of secretory autophagy in cancer remains obscure and needs to be further explored.

## 3. Extracellular Vesicles

### 3.1. EVs Biogenesis and Secretion Mechanisms

Extracellular vesicles (EVs) represent a great interest in the field of cell-cell communication. Indeed, several populations of EVs are secreted by cells under various conditions [15,18,19]. As so, apoptotic cells secrete apoptotic bodies of about 1 to 5 µm wide, which contain information from the dying cells [18]. On the other hand, microvesicles (MVs) have a smaller size range (100 nm to 1 µm) and are secreted by plasma membrane budding [18]. The smallest population of secreted EVs are exosomes (50 to 150 nm in diameter), which are also known for being involved in cell-cell communication by carrying lipids, proteins or RNA as pieces of information towards towards the neighbouring cells [14,15,16,17]. Unlike apoptotic bodies and MVs, exosomes have an endosomal origin. During their maturation, endosomes form intra-luminal vesicles (ILVs) by invagination of their membrane, generating multi-vesicular bodies (MVBs) which fuse with the plasma membrane to release exosomes in the extracellular compartment [20,28].

The formation of MVBs for exosomes biogenesis can be dependent or independent on the endosomal sorting complex required for transport (ESCRT) machinery. The ESCRT-dependent pathway involves four successively operating protein complexes (ESCRT-0, I, II and III), and their associated complex Vps4 [20,28]. ESCRT-0 first recognizes and catches ubiquitinated cargo in clathrin-coated domains through its ubiquitin-binding domains (UBDs), and binds PI3P on the endosomal membrane, thus initiating the ESCRT machinery for cargo sorting and MVB formation [21]. ESCRT-0 is also able to bind the ESCRT-I complex, notably composed of TSG101, that also recognizes ubiquitinated cargo, thus pursuing its sorting and providing membrane budding. ESCRT-I recruits ESCRT-II on the membrane of future MVBs to help budding as well as recruiting and activating ESCRT-III. This last complex needs the help of accessory proteins such as Alix to make it functional. Concomitantly, ESCRT-III interacts with the AAA ATPase Vps4 which is mandatory for the final step of MVB formation, by providing de-ubiquitination of the cargo and ESCRT-III detachment, thus inducing the whole ESCRT complex to unbind the endosomal membrane [21,22,23,24,25,26,28]. As proof of their implication in exosomes biogenesis, some ESCRT-associated proteins such as Tsg101 (ESCRT-I member) or Alix are found in exosomes after their release [27].

Although deletions in the ESCRT pathway impair cargo recognition and sorting, they do not completely inhibit the formation of MVBs in mammals, suggesting that ESCRT machinery works in coordination with other processes, notably involving tetraspanin proteins (CD63, CD81), lipids such as ceramide, or heat shock proteins [23,81,82,83,84]. Indeed, Stuffers et al. [81] reported that deleting some ESCRT actors impaired the morphology and size of the ILVs within MVBs, suggesting that ESCRTs remain important for their formation. However, a recent model suggests that the ESCRT machinery is particularly important for a specific cargo sorting before entering ILVs [23]. Thus, a complementary mechanism for membrane deformation to englobe the ESCRT-recruited cargo is needed, and this role may be played by the presence of particular lipid domains on the endosomal membrane, notably containing ceramide [23,81,85]. It was indeed shown that inhibition of the ceramide formation process led to defects in releasing proteolipid protein-containing exosomes, whereas ESCRT inhibition did not affect such a release, suggesting that their secretion was ESCRT-independent [81]. Tetraspanins, of which some are considered exosome markers (such as CD63 or CD81), also seem to be involved in specific ESCRT-independent exosome release [83,85]. Finally, it is supposed that heat-shock proteins such as Hsc70 can play a role in such ESCRT-independent processes by their ability to deform membranes [84,85]. These findings may suggest that the ESCRT machinery is not mandatory in the formation of MVBs and that some ESCRT-independent actors operate in the releasing of EVs, and particularly exosomes, based on their specific content, as well as the fact that cells may secrete different types of EVs regarding their structure and content based on the different ways MVBs may be formed [26].

**Figure 2:**
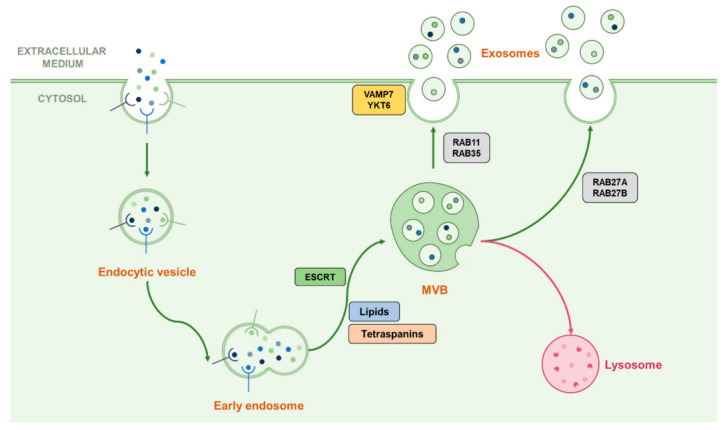
Representation of the classical biogenesis and secretion machinery of exosomes. Early endosomes can follow their maturation by forming ILVs contained in MVBs by using multiple ways dependent or not from the ESCRT machinery. Once the MVB formed, it can be addressed to the lysosome in order to degrade its cargo or ILVs can be released in the extracellular medium as exosomes. The transport toward the cell membrane is proceeded by Rab-GTPases than can vary following the cell type or the cellular conditions, and the fusion with the plasma membrane can be driven by the SNAREs VAMP7 and YKT6.

Once MVBs are formed, they require transport toward the cell membrane in order to release exosomes in the case of secretory vesicles, or toward the lysosome for the degradation of their cargo. Such transport involves the small Rab-GTPases family which is known to be implicated in vesicular trafficking processes, here notably represented by Rab7, 11, 27A/B and 35, which both play a different role in exosome fate [14,28,29,30]. For example, Rab7, which is known for transport toward the lysosome in autophagy machinery, also operates in the regulation of the secretion of exosomes containing syndecan and syntenin in breast cancer cells but not in HeLa cells [29,31]. Rab11 was shown to promote the fusion of MVBs with each other (homotypic fusion) and is often associated with Rab35 in early endosomal recycling processes [30,32]. Rab27A and Rab27B, which are known for their late endosomal recycling ability, have also been shown to play both common and different roles by providing MVB docking to the cell membrane depending on the cell type [29,33]. After the transport of MVBs to the cell membrane, they fuse with each other through the action of SNARE proteins such as VAMP7, shown as implicated in this process in leukemic cells, or YKT6 which operates in the secretion of Wnt proteins-containing exosomes [14,28,34,35]. Since those many actors play different roles in different steps of the exosomal secretion process, which seem to be specific according to the cell type, this may partly explain the variation of their content [26]. This machinery is summarised in Figure 2.

### 3.2. Implications in Cancer Development: Increasing Interest in This Field

Initially described as “trash”, the view on these vesicles (especially exosomes) has been modified. EVs are implicated in intercellular communication by carrying information from the sending cells [14,15,16,17,86]. Thus, such a process constitutes an obvious means by which cancer cells might communicate with their environment. As such, exosomes are able to deliver their cargo in recipient cells by activating various signalling pathways that include stem cell maintenance and renewal [87]. Hence, the study of EVs secretion in the field of cancer research has aroused a great interest in recent years, especially with regards to their roles in cancer development.

EVs and particularly exosomes have been notably shown to play various roles in many cancer-associated mechanisms. First, EVs are used as transmitters of the oncogenic receptor EGFRvIII between tumour cells in glioblastoma [88]. Our team has reported that TrkB-containing exosomes are implicated in the transfer of aggressiveness in glioblastoma cells [89]. On the other hand, tumour cells are able to communicate with surrounding non-tumour cells by sending EVs containing miRNAs, proteins and oncogenic factors, thus promoting tumour invasion and angiogenesis [90,91,92,93]. In order to promote tumour invasion, EVs can also play a role in extracellular matrix remodelling and epithelial/mesenchymal transition (EMT) that represents the first step towards metastatic migration [36,37,38]. Moreover, EVs are involved in cancer therapy resistance by controlling the tumour microenvironment integrity towards drugs and promoting CSC-like behaviour such as quiescence and EMT [94,95].

In CRC, it was notably demonstrated that exosomes were implicated in the transfer of mutant KRAS from mutant cells towards non-mutant KRAS cells, thus providing an increase in tumour growth in vitro [39]. Similarly, *Apc* mutation was shown to enhance EVs secretion by CRC tumour cells within 3D organoids [40]. As with other cancers, EVs are used as messengers between tumour cells and stromal cells to promote tumour invasion [41]. It is noteworthy that cell differentiation seems to impact EV secretion and their contents, suggesting that their role may vary according to the differentiation state of the sending cells [42]. Since there is a lack of specific CSC markers, it is interesting to consider the machinery for secretion of EVs as a marker of cancer development. However, this process is also used by stromal cells and thus cannot be considered as a therapeutic target. Since vesicular trafficking seems to be closely related to the secretory autophagy process, and considering that these processes are both implicated in cancer development-associated mechanisms, this potential overlap may nevertheless be considered a potential pathway for further investigation.

## 4. Autophagy and EVs Trafficking Cooperation: Which Actors

### 4.1. Common Proteins and Pathways

Although the crosstalk between autophagy and vesicular trafficking remains obscure, some clues may point towards the role of common proteins in such an interaction. Among those, as previously described, Rab-GTPases are known to be implicated in both processes. For instance, Rab7 was notably found as operating both in the autophagy pathway and EV trafficking according to a database comparison, as well as Rab11A [14]. In the same way, Rab8A, which is known for its implication in unconventional secretory autophagy by promoting the guiding of autophagosomes toward the cell membrane, was also shown to be involved in the exosomal secretion of Annexin A2 in coordination with Rab11 and Rab27A by enhancing amphisome/plasma membrane fusion [12,71]. Furthermore, in the same study, autophagy-related proteins were also shown to play their part in the secretion of Annexin A2-contatining exosomes, such as ATG5 and LC3B, further supporting the hypothesis of an autophagy/EV-trafficking overlap [12,13].

LC3B was recently found to be implicated in the secretion of specific RNA-binding proteins (RBPs) and small nucleolar RNAs (sno-RNAs) within EVs [96]. This mechanism seems to involve an ATG7-dependent pathway and represents an unconventional route for ATG-dependent EVs secretion. This process, called “LC3B-dependent EV loading and secretion” (LDELS), requires the interaction between LC3B and FAN (neutral sphingomyelinase activation associated factor) that acts as a regulator of nSMase2, which produces ceramides required for the inward budding of vesicles from the MVB [96]. Curiously, autophagy-related proteins may also play autophagy-independent roles in other processes, as demonstrated for ATG5. Indeed, a loss in the expression of ATG5 or its complex partner ATG16L1 led to a decrease in exosome secretion, which was not found after deleting *Atg7* [97,98]. It was further suggested that ATG5 was involved in regulating the pH within MVBs by recruiting LC3B that associates with the proton pomp V_1_V_0_-ATPase, which controls MVBs acidification, thus preventing their lysosomal degradation. Furthermore, the ATG5-ATG16L1 complex seems to be implicated in promoting metastases and migration in breast cancer cells by releasing exosomes [97,98]. Another example of ATG protein involvement in EV biogenesis mechanisms may be ATG9, which was shown to be required for the formation of ILVs since loss of its expression reduced autophagic flux and ILVs production in *Drosophila melanogaster* [99]. Similarly, ATG3 and ATG12, known for their role in autophagosome maturation and LC3B lipidation respectively, were shown to interact with the ESCRT-associated protein Alix, thus suggesting their implication in many related processes such as autophagic flux, endosomal trafficking and exosomes biogenesis [100]. The autophagy-initiating class III PI3K complex has also been suggested as playing a part in exosome biogenesis and release, since the inhibition of Beclin-1 expression led to a decrease of chronic myeloid leukaemia cells-secreted exosomes [101]. However, the role of this complex in exosome biogenesis requires further investigation, since it is supposed to play various roles following its regulation by many proteins, as reviewed by Xu et al. [13]. On the other hand, other proteins such as HSPA8, HSP90 and VCP, introduced as key autophagy-associated proteins by Galluzzi et al. [102], were identified in EV-related experiments, although further information is needed concerning their role in vesicular trafficking and release [14]. As SNAREs such as SEC22B are implicated in vesicular transport and fusion with the cell membrane, they can be supposed to be part of the autophagy/EV-trafficking crosstalk, though more information is needed [103]. Since different proteins, complexes and or pathways seem to be commonly used in autophagy-associated and EV-associated mechanisms, it seems fair to think that these actors operate around common organelles, as elaborated below.

### 4.2. An Important Common Organelle: The Versatile and Misunderstood Amphisome

Among the different organelles and structures that intervene in each pathway, amphisomes appear as real crossroads between autophagy (more particularly secretory autophagy) and EV biogenesis and release. Amphisomes were initially considered as exclusive degradative organelles. For instance, increased autophagy under stress conditions or Bafilomycin A treatment, leading to increased autophagosome-MVBs fusion, was associated with fewer secreted exosomes in erythroleukemic cells, suggesting an autophagic recycling of MVBs instead of their release, thus supporting the close relationship between autophagy and exosome release via amphisomes [13,104]. Also, alteration in exosome biogenesis could be restored after autophagy inhibition, supporting the hypothesis that MVBs can be recycled in a lysosome-dependent manner rather than giving rise to exosomes release [79]. However, amphisomes were also shown to participate in non-degradative mechanisms as the previously described intervention of the autophagy proteins LC3B and ATG5 in the secretion of Annexin A2-containing exosomes requires the fusion of amphisomes with the plasma membrane [12]. Similarly, amphisomes were shown to be involved in a secretory pathway for mucin-containing vesicles in murine intestinal cells, requiring the intervention of LC3B, RAB7 and RAB11, thus supporting a potential secretory role for amphisomes fate [105]. Some points remain uncertain regarding the fate of autophagosomes toward a fusion with lysosomes or MVBs. Indeed, the secretion of IL-1β requires the fusion of autophagosomes with MVBs, supporting the unconventional autophagy-dependent secretory pathway model proposed by Dupont et al. [71] (while it was not determined if such a fusion led to the formation of an amphisome) [105].

**Figure 3:**
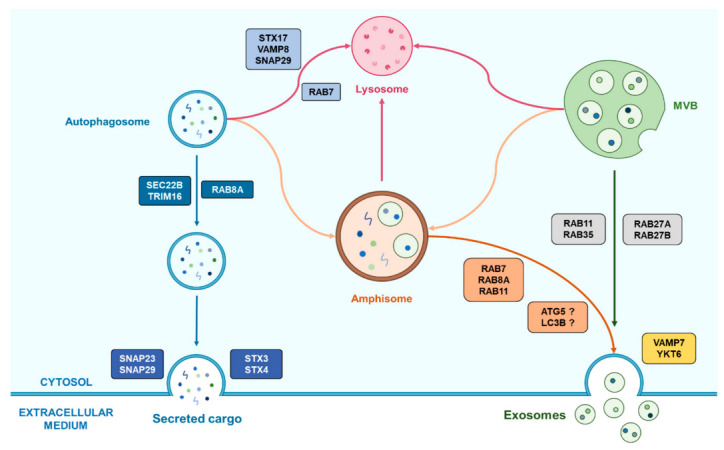
Representation of the emerging cooperation between autophagy and exosomes biogenesis helped by the amphisome. Autophagosomes can fuse with MVBs in order to form amphisome which have two possible fates following the cell conditions. First, the amphisome is able to fuse with a lysosome for the degradation of its cargo and its recycling. Alternatively, amphisomes can also be implicated in secretory processes by the means of Rab proteins that can vary according to the cell type. This last process may also require autophagy-related proteins which role remains unclear.

The roles of amphisomes seem to vary according to the different conditions a cell can undergo, which correlates with the way autophagy and the secretion of small EVs such as exosomes are modulated following these conditions. Further information is required to better understand this potential hub that amphisomes may represent between autophagy and exosome biogenesis and release, and to enlighten how autophagy and exosomes really interact. An overview of the known roles of amphisomes in such an interaction are presented in Figure 3.

Since cells must survive in stress conditions by using autophagy and/or EVs secretion as discussed earlier, it seems interesting to investigate whether their cooperation may occur in cancer development. Even though little is known about this, some studies have already focused their attention on such an eventual interaction. For example, the application of rotenone in breast and prostate cancer stem cells, which imitates a respiratory stress by altering mitochondrial activity, resulted in increased autophagy and exosome release, suggesting a collaboration of these processes in the stress response [106,107]. The hypothesis concerning their cooperation in responding to cellular stress is supported by an observation of increased autophagy and EVs secretion after drug treatments and in chemo-resistant cancer cells, although there is no clear confirmation of their cooperation [108,109,110]. These stress response mechanisms remain unclear and need further investigation, because it is not known yet if such observations are the result of a real coordination between autophagy and EVs biogenesis and release, or whether it only represents a way cells respond to stress conditions.

## 5. Conclusions

Autophagy and the secretion of small EVs, and particularly exosomes, seem to be closely related. Indeed, the discovery of a secretory pathway for autophagy tends to support the hypothesis of a link between these two processes. Moreover, common actors such as the Rab-GTPases and SNARE protein families were already shown to operate in both mechanisms. Since they are implicated in cancer development in different ways, studying the interactions between autophagy and the secretion of EVs, and especially exosomes, may be of great interest in the pursuit of a better understanding of some of the more obscure parts of cancer development that remain elusive.
